# Down-regulation of tenascin-C inhibits breast cancer cells development by cell growth, migration, and adhesion impairment

**DOI:** 10.1371/journal.pone.0237889

**Published:** 2020-08-20

**Authors:** Dariusz Wawrzyniak, Małgorzata Grabowska, Paweł Głodowicz, Konrad Kuczyński, Bogna Kuczyńska, Agnieszka Fedoruk-Wyszomirska, Katarzyna Rolle

**Affiliations:** 1 Department of Molecular Neurooncology, Institute of Bioorganic Chemistry of the Polish Academy of Sciences, Poznan, Poland; 2 NanoBioMedical Centre, Adam Mickiewicz University, Poznan, Poland; 3 Laboratory of Subcellular Structures Analysis, Institute of Bioorganic Chemistry of the Polish Academy of Sciences, Poznan, Poland; Thomas Jefferson University, UNITED STATES

## Abstract

Tenascin-C (TNC) is an extracellular matrix (ECM) glycoprotein that plays an important role in cell proliferation, migration, and tumour invasion in various cancers. TNC is one of the main protein overexpressed in breast cancer, indicating a role for this ECM molecule in cancer pathology. In this study we have evaluated the TNC loss-off-function in breast cancer cells. In our approach, we used dsRNA sharing sequence homology with *TNC* mRNA, called ATN-RNA. We present the data showing the effects of ATN-RNA in MDA-MB-231 cells both in monolayer and three-dimensional culture. Cells treated with ATN-RNA were analyzed for phenotypic alterations in proliferation, migration, adhesion, cell cycle, multi-caspase activation and the involvement in epithelial to mesenchymal transition (EMT) processes. As complementary analysis the oncogenomic portals were used to assess the clinical implication of TNC expression on breast cancer patient’s survival, showing the TNC overexpression associated with a poor survival outcome. Our approach applied first in brain tumors and then in breast cancer cell lines reveals that ATN-RNA significantly diminishes the cell proliferation, migration and additionally, reverses the mesenchymal cells phenotype to the epithelial one. Thus, TNC could be considered as the universal target in different types of tumors, where TNC overexpression is associated with poor prognosis.

## Introduction

The tumor microenvironment is composed of the surrounding stromal cells, such as endothelial cells in blood vessels, immune cells, fibroblasts, and the extracellular matrix (ECM) [1, 2]. During carcinogenesis is often perturbed and deregulated, while during embryonic development is strictly controlled to maintain homeostasis [3]. In tumors, the composition of the ECM differs from that of normal tissue and enables new interactions that affect the function of cancer cells and are critical in modulating invasion associated with cell migration and growth. The tumor-associated ECM presents several tumor-associated antigens that are generally more abundant and possibly more stable than those of the cell surface [4–6]. Consequently, these proteins represent possible valuable targets for tumor imaging and therapy [4, 5]. ECM proteins such as fibronectin (FN) and tenascin have isoforms that are expressed in a tissue specific manner generated by alternative splicing of their primary transcripts. One of the most consistent isoform changes in the ECM of many tumors is the up-regulation of the glycoprotein, tenascin-C (TNC). TNC alongside tenascin-X (TNX), tenascin-R (TNR) and tenascin-W (TNN) are members of, well conserved among vertebrates, tenascin family (TN) [7–12]. Numerous isoforms of TNC can be produced through alternative splicing of nine fibronectin type III regions between repeats 5 and 6 at the pre-mRNA level. There is a considerable amount of literature on the contribution of different splicing-dependent TNC domains in specific biological functions [13]. Changes in the TNC isoforms expression pattern have been then described in a number of malignancies, and their nature appears to be tumor-type specific. Recent studies have demonstrated that some splice variants are specific to diseased tissues [14–16]. In breast tissues, expression of two TNC variants, one containing domain D and the other both B and D, was found to be associated with invasive phenotype [17]. TNC promotes cell migration, angiogenesis, inhibit focal contact formation, and also act as a cell survival factor [18–22]. Its importance was found in the development and progression of different types of neoplasm, including: colon and breast cancer, fibrosarcoma, lung cancer, melanoma, squamous cell carcinoma, bladder cancer, and prostatic adenocarcinoma [23, 24]. TNC is also highly expressed in high-grade gliomas which correlates as well with the invasiveness of glioma cells [25–27]. In the brain, it is important for the development of neural stem cells [28, 29] and moreover is suspected to be a potential marker for glioblastoma multiforme (GBM) stem cells (GSC) [30].

Previously, we have shown that TNC is overexpressed in GBM and can be a good target in RNAi approach. With 164-nt long dsRNA complementary to the mRNA of TNC, which we called ATN-RNA, we conducted the experimental therapy for GBM patients [25]. The discovery that TNC presents a dominant epitope in glioblastoma prompted us to investigate the potential of ATN-RNA to block the TNC expression and its effect on the growth of human breast cancers, where TNC overexpression was also established and linked with the highest malignancy, invasion capability and metastasis ability. This view is supported by Mock et al., who showed that GBM patients with antibodies against the EGF-like repeats of TNC (antibody target: VCEDGFTGPDCAE) have a significantly better prognosis than other patients [31]. Thus we assumed, that in the light of the satisfactory results of brain tumors experimental therapy, breast cancer could be the next possible object of interest to establish the ATN-RNA approach.

Here, we demonstrate that ATN-RNA approach can be successfully used in breast cancer cells, impairing the basic hallmarks of tumor cells. With the performed analysis of proliferation, migration rate, multi-caspases induction pathway, cell cycle analysis, spheroids viability and the involvement of TNC in EMT induction, we have then interrogated the impact of TNC on breast cancer growth showing its potency to be also the promising therapeutic target in breast cancer treatment.

## Results

### Oncogenomic *in silico* analysis reveals the TNC correlation with poor survival of breast-cancer patients

To look deeper into the TNC function we performed the analysis of genome-wide breast cancer data with available oncogenomic portals such as GEPIA, the Human Protein Atlas, cBioPortal, and PPISURV. Based on the status of three important receptors conventionally used for breast cancer subtyping, i.e., estrogen receptor (ER), progesterone receptor (PR), and human epithelial receptor 2 (HER2), breast cancer is classified as luminal A, luminal B, HER2 positive and triple-negative (“basal-like”). Triple-negative and HER2-overexpressing breast cancer yields a poor patient prognosis because of a high incidence of metastases, disease progression, and resistance to current chemotherapy regimens [32]. We first compared the expression level of tenascin-C in 4 subtypes of breast cancer using GEPIA program (Fig A in S1 File). mRNA level of TNC were higher in triple-negative and HER2 subtypes compared to the luminal A and luminal B subtypes, which have a better prognosis for patient survival. Therefore, we chose MDA-MB-231 cells as a model for *in vitro* experiments because it is the most invasive cell line from breast cancer models. MDA-MB-231 cell genome clusters with the basal subtype of breast cancer. Since the cells also lack the growth factor receptor HER2, they represent a good model of triple-negative breast cancer [33]. What is important, Adams et al. [17] showed that only invasive cell lines such as MDA-MB-231 or MDA-MB-468 express tenascin-C, whereas the tumor cell lines with a low invasive capacity, MCF-7 and T47D do not.

**Fig 1:**
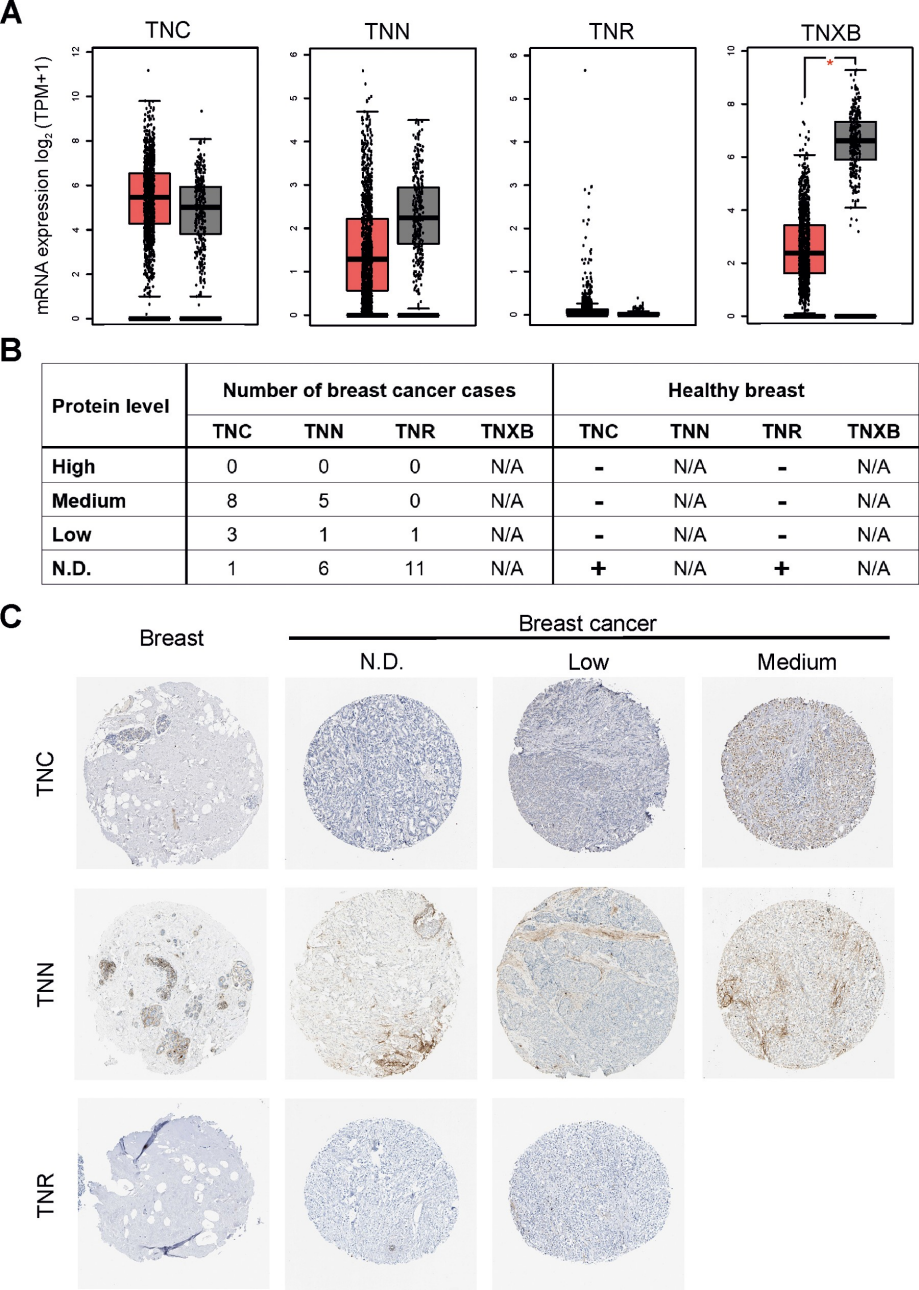
Tenascin is highly expressed in breast invasive carcinoma (TCGA-BRCA). **(A)** Messenger RNA levels of TNC (tenascin-C), TNN (tenascin-W), TNR (tenascin-R), TNXB (tenascin-X) genes in 1085 specimens from patients with invasive carcinoma of the breast (*vs*. 291 non-tumor samples). RNA sequencing data were retrieved from the database of TCGA and analysed using the GEPIA (Gene Expression Profiling Interactive Analysis) online web server (http://gepia.cancer-pku.cn/). The red boxes represent cancer specimens, grey boxes represent healthy breast specimens. Significance value: * *P* < 0.05. **(B)** Summary of tenascin expression patterns in breast cancer tissues and healthy breast determined by immunohistochemical staining. Data were retrieved from the Human Protein Atlas database. Case numbers of invasive breast cancer are shown. N/A: not available; N.D.: not detectable. In “Healthy breast” column “+” means that TNC and TNR are not detected in non-tumor samples. Results in normal breast are based on immunohistochemical staining of a single sample. **(C)** Representative images of immunohistochemical staining for TNC, TNN, and TNR in breast (healthy tissue) and invasive breast carcinoma specimens. The images shown here are of the tissue sections from tissue microarray arrays (TMAs) stained with appropriate antibodies; TNC–CAB004592, TNN–CAB010907, TNR–CAB022343. All the images were taken at 200× magnification.

As a next step we compared the expression levels of tenascin genes (*TNC*, *TNN*, *TNR*, *TNXB*) in invasive breast cancer using GEPIA program (Fig 1A). mRNA level of TNC was highly expressed in breast cancer tissue (BRCA). Interestingly, expression levels of *TNN* and *TNXB* were significantly lower in breast cancer tissues (Fig 1A). There was no significant difference in *TNR* gene expression between breast cancer and non-tumor tissues.

We also examined the expression of tenascin proteins in normal and malignant tissues by querying data from the Human Protein Atlas. TNC in most cases and partly TNN were expressed at medium levels, whereas TNR was not detected (Fig 1B and 1C). TNC and TNR levels were undetectable in samples from normal breast (adipocytes, glandular and myoepithelial cells). Taken together, our results demonstrate that mRNA and protein levels of TNC is relatively higher in invasive breast cancer tissues than those in normal tissues.

The cBioPortal analysis enabled to look for the mutations in the *TNC* gene. It appeared that *TNC* gene mutations measured for 2051 breast cancer patients, are present as somatic mutation only in 11 cases (0.5%). Since these mutations seem to be irrelevant for breast cancer, we did not perform any further analysis.

With PPISURV portal we looked through the transcriptomic data to correlate the TNC expression with the different clinical parameters, such as survival or prognosis of the cancer.

**Fig 2:**
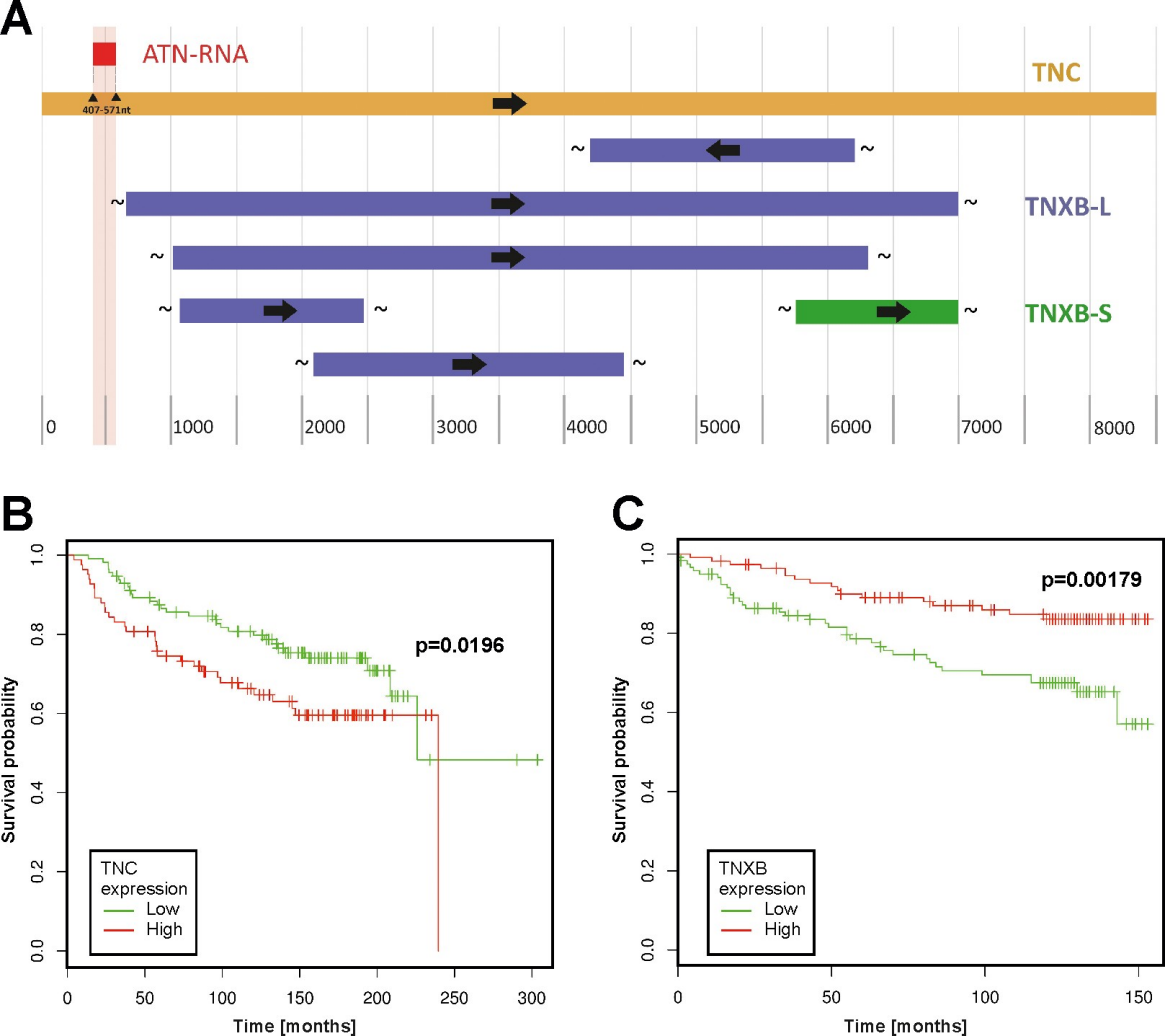
The oncogenomic analysis of the survival association with tenascin-C and tenascin-X in breast cancer. **(A)** Sequence alignment of tenascin-C versus tenascin-X with their relation to ATN-RNA. The sequence alignment analysis clearly show the ATN-RNA matching exclusively to the TNC sequence (100% identity). Relation of TNC **(B)** and TNXB **(C)** gene expression to survival of breast cancer patients. Survival analysis performed with the use of a dataset (breast cancer; GEO: GSE7390 and GSE3494) deposited in and tools available from the PPISURV web portal. TNXB-L- long transcription variant; TNXB-S- short transcription variant.

As the initial step of analysis we performed the alignment of TNC and other proteins from the tenascin family, such as tenascin-X (TNX) looking for the homology between these two proteins. At the top of that we made the analysis of the homology between the TNC and TNX with the relation to ATN-RNA sequence. The alignment of these two ECM proteins shows that they share only limited number of nucleotides (query cover 5% and 22%, for short and long transcriptional variants in the protein N-terminus region, respectively. The sequence alignment analysis clearly show the ATN-RNA matching exclusively to the TNC sequence (100% identity) (Fig 2A).

PPISURV analysis based on the Kaplan-Meier statistics showed very clearly the strong correlation with TNC expression and patients survival. The high expression level has a great impact on the shorter survival for the patients, thus suggesting also that TNC can be also considered as the prognostic factor for breast cancers (*P* = 0.0196) (Fig 2B). At the same time, we analyzed also the available data for the TNX. The results showed an inverse correlation of *TNXB* mRNA expression and survival (*P* = 0.00179) (Fig 2C). Analysis was carried out on the group of patient N = 196 for *TNC* and N = 232 for *TNXB*.

### ATN-RNA mediates the down-regulation of TNC mRNA and protein expression in breast cancer cells

**Fig 3:**
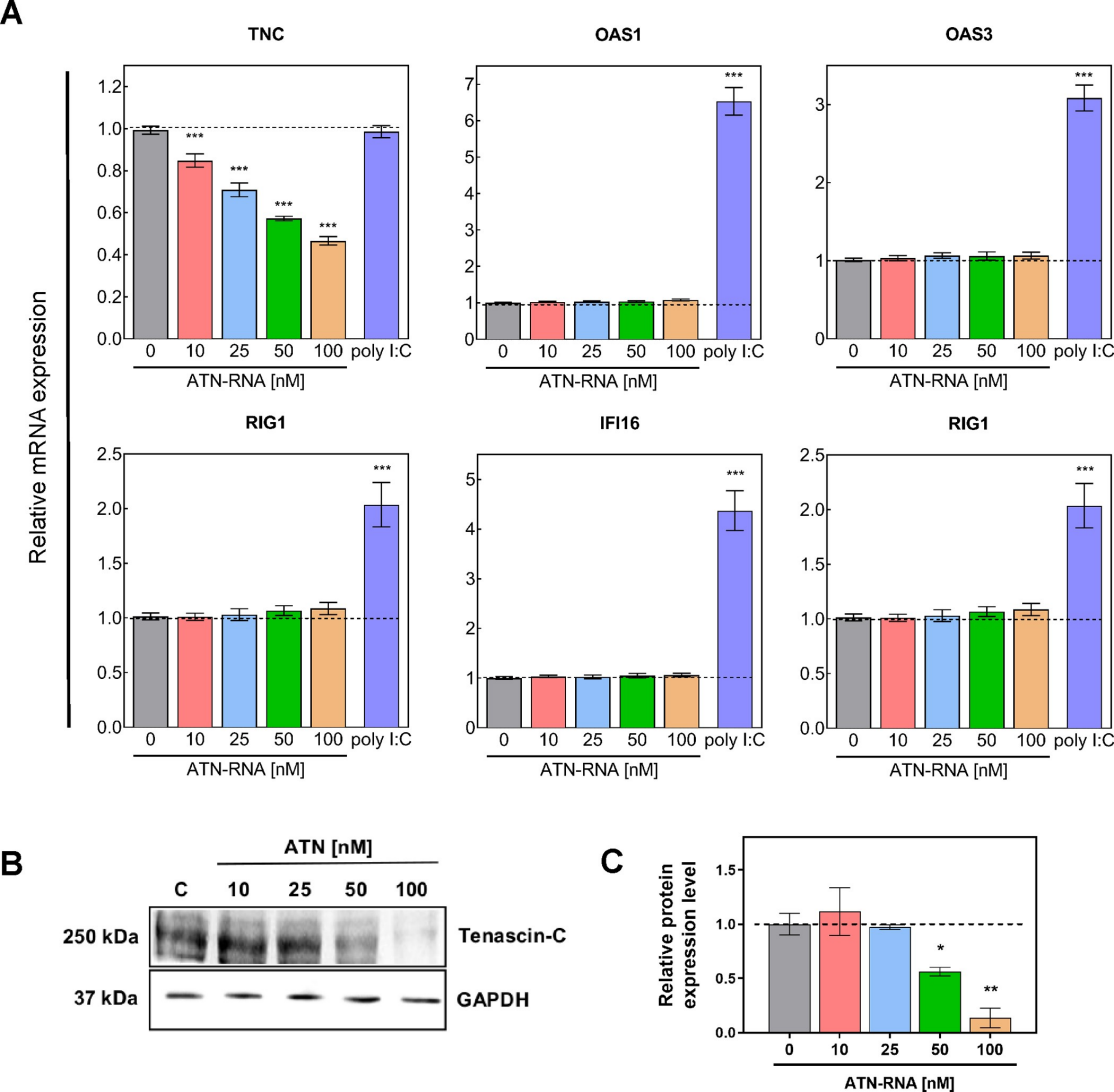
Expression level of TNC and immune response genes after ATN-RNA treatment in MDA-MB-231 cell line. **(A)** Relative expression level of the expression of *TNC*, *OAS1*, *OAS3*, *RIG1*, *IFI16* and *TLR3* established by qRT-PCR. Relative expression was calculated using the–ΔΔC_p_ method. Statistical evaluation of ATN-RNA *versus* scrambled siRNAs (C-control) cells was performed using one-way ANOVA followed by Tukey’s post-hoc test. Effect of poly I:C (100 μg/ml) on immune response genes (*OAS1*, *OAS3*, *RIG1*, *IFI16*, *TLR3*) in the figure presented as purple bars. The results for *HPRT*-normalized expression of mRNA are expressed as fold change of target gene expression relative to the control (without poly I:C treatment, which is defined as 1). **(B)** The protein expression levels of *TNC* and *HPRT*. **(C)** Western blot analysis reveals efficient TNC silencing in MDA-MB-231 cells with ATN-RNA, compared to cells treated with siRNAs (C-control). The data represents the means ± SD from 3 independent experiments. Significance value: * *P* < 0.05, ** *P* < 0.01, *** *P* < 0.001.

To achieve down-regulation of TNC expression in breast cancer cell line, transfection with various concentration of ATN-RNA (10; 25; 50 and 100 nM) was performed. 48 h after transfection the expression level of TNC was examined by qRT-PCR analyses. Significant down-regulation of TNC mRNA expression was observed compared to control treated with scrambled RNA. The level of TNC was decreased from 15% at a concentration of 10 nM ATN-RNA up to 55% for cells treated with 100 nM ATN-RNA in comparison to the control (*P* < 0.001) (Fig 3A).

The qRT-PCR analysis was also supported by direct analysis of the protein expression level. We have observed already a 44% decrease in TNC protein expression upon 50nM ATN-RNA treatment. The highest concentration (100nM) used led to the dramatic drop of the protein expression measured as the 86% of the decrease (Fig 3B and 3C). These observations were fully consistent with relative TNC expression level measured by qRT-PCR.

### Interferon response to ATN-RNA

To establish interferon induction in breast cancer MDA-MB-231 cultured cells, we looked for interferon stimulated genes (ISG) including: *OAS1*, *OAS3*, *RIG1*, *TLR3* and *IFI16* genes. The analysis was carried out with the qRT-PCR (Fig 3A). Changes after ATN-RNA measured by qRT-PCR were not significant, as shown basically for all of the genes in the concentration range of 10–100 nM. In parallel, a synthetic form of dsRNA, poly(I):poly(C) (poly I:C), was transfected as a positive control. Poly I:C has been used extensively as a TLR3 ligand to induce antiviral response [34–36]. We showed that transfection with poly I:C (100 μg/ml) efficiently induced the expression of immune response genes (*OAS1*, *OAS3*, *RIG1*, *IFI16*, *TLR3*) in MDA-MB-231 cells. This enhancement in mRNA expression was 2–7-fold higher in poly I:C treated cells than in untreated cells (Fig 3A, purple bars).

### TNC knockdown inhibits cells proliferation and leads to the changes in migration rate and adhesion potential of breast cancer cells

**Fig 4:**
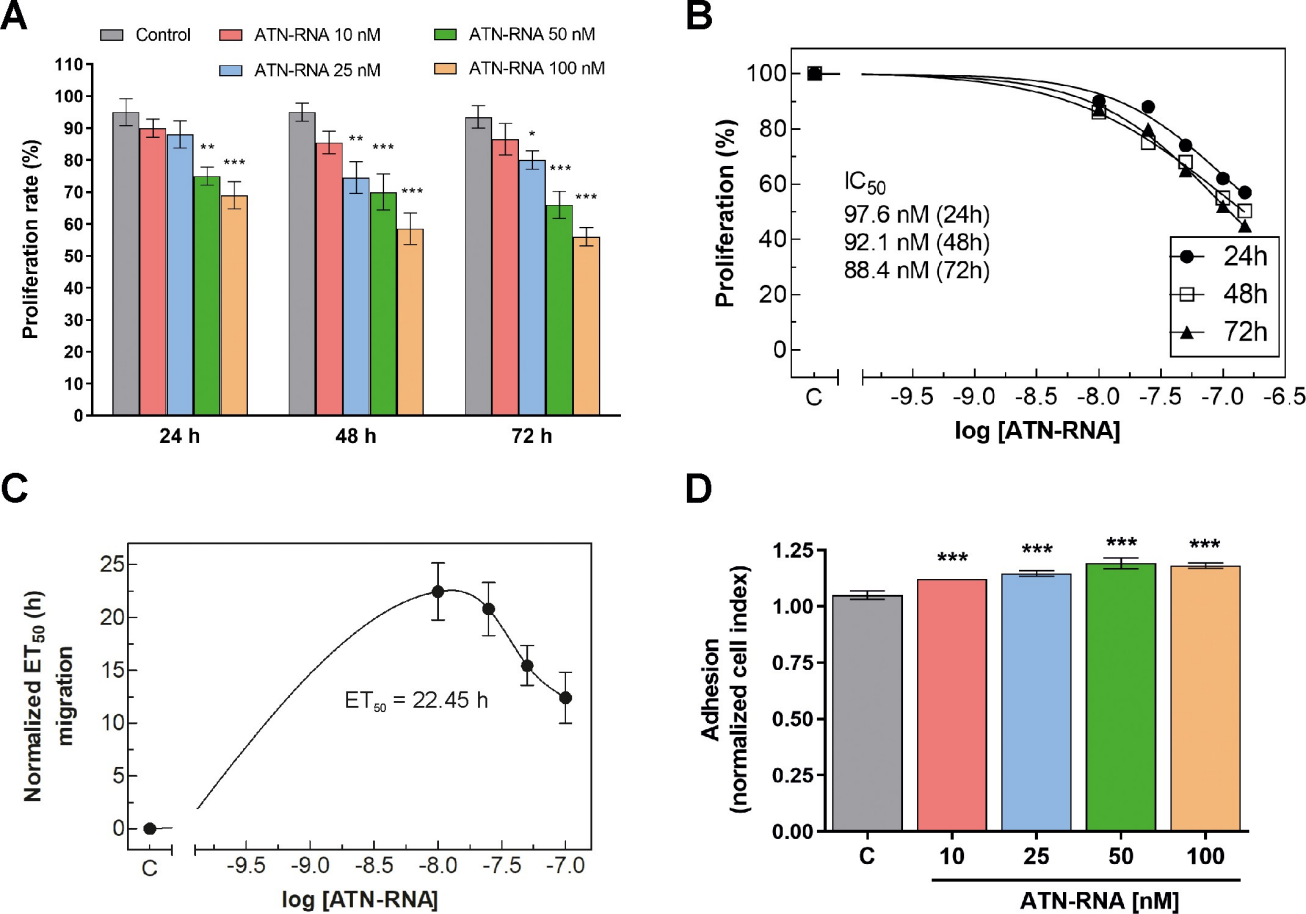
Activity of ATN-RNA in proliferation, migration and adhesion. Proliferation of breast cancer in culture was monitored in real-time using *xCELLigence* system. **(A)** Impedance was recorded every 15 min, but to improve the clarity of the graphs only every fourth readout was plotted. Data show the mean ± SD of three independent measurements. **(B)** Dose-dependent effects of ATN-RNA on proliferation was evaluated using non-linear regression by fitting experimental values to sigmoidal, bell-shaped equation. **(C)** Migration of MDA-MB-231 cancer cells was studied using *xCELLigence* system. Serum-depleted cells were transfected with increasing concentrations of ATN-RNA (from 10 to 100 nM) or scrambled siRNAs (C-control). Impedance (CI values) of each experimental condition was recorded over time, plotted against time, fitted to four-parameter logistic non-linear regression model and ET_50_ was calculated for each ATN-RNA concentration to generate dose-response curves. ET_50_ value was normalized to the data obtained for untreated cells and plotted as normalized half maximal effective time (ET_50_) of cell migration against ATN-RNA concentrations. **(D)** Adhesion of MDA-MB-231 cell line was observed in real-time using *xCELLigence* system. Graph shows the final impendence values minus the initial values for the respective samples. Differences between CI values for ATN-RNA treated and control cells were statistically evaluated using one-way ANOVA followed by Tukey’s post-hoc test (symbols above the bars). Significance value: *** *P* < 0.001 compared to cells treated with scrambled siRNAs (C-control).

In order to investigate the involvement of TNC on breast cancer cells proliferation, MDA-MB-231 cell line was treated with ATN-RNA and the real-time cell proliferation assay was performed. The cells ability to proliferate was measured for 72 h. We have noticed time- and concentration-dependent decrease in proliferation rate. The most effective concentration of ATN-RNA was 100 nM, with decrease from 32–45% after 24 and 72 h, respectively (Fig 4A). Noteworthy, 25 nM and 50 nM of ATN-RNA was already sufficient concentration for the efficient inhibition of breast cancer cells proliferation. The dose-dependent effect of ATN-RNA in MDA-MB-231 proliferation potential resulted in standard sigmoidal dose-responses with IC_50_ of 97.6 ± 8.2 nM after 24 h, 92.1 ± 7.8 nM after 48 h and 88.4 ± 5.2 nM after 72 h of treatment (Fig 4B).

To get more insight into the down-regulation of TNC expression by ATN-RNA on the mobility of breast cancer cells, real-time measurements of migration was carried out. We found that down-regulation of TNC expression by ATN-RNA significantly impaired the cell migration in breast cancer cell lines (Fig 4C). The results were quantitatively assessed during 72 h of experiment and showed that MDA-MB-231 cells transfected with ATN-RNA had the lowest motility beginning from 12 h post transfection. It was established that ATN-RNA delayed the migration of MDA-MB-231 cells by 22.45 ± 2.7 h, 20.80 ± 2.5 h, 15.44 ± 1.9 h and 12.40 ± 2.5 h with 10, 25, 50 and 100nM concentration, respectively. Notably, the most effective concentration which affected the migration potential of the cells was 10nM. When compared to the control, the observed delay was 22.45 ± 2.7 h.

Since TNC is implicated also in cell-matrix attachment, we further looked at the adhesion ability of ATN-RNA treated cells. The cells were conducted to real-time adhesion assay with *xCELLigence* system. Compared with the controls, TNC knockdown resulted in increased cells adhesion 15% on average (Fig 4D).

### TNC promotes apoptosis and is involved in cell cycle regulation in breast cancer cells

**Fig 5:**
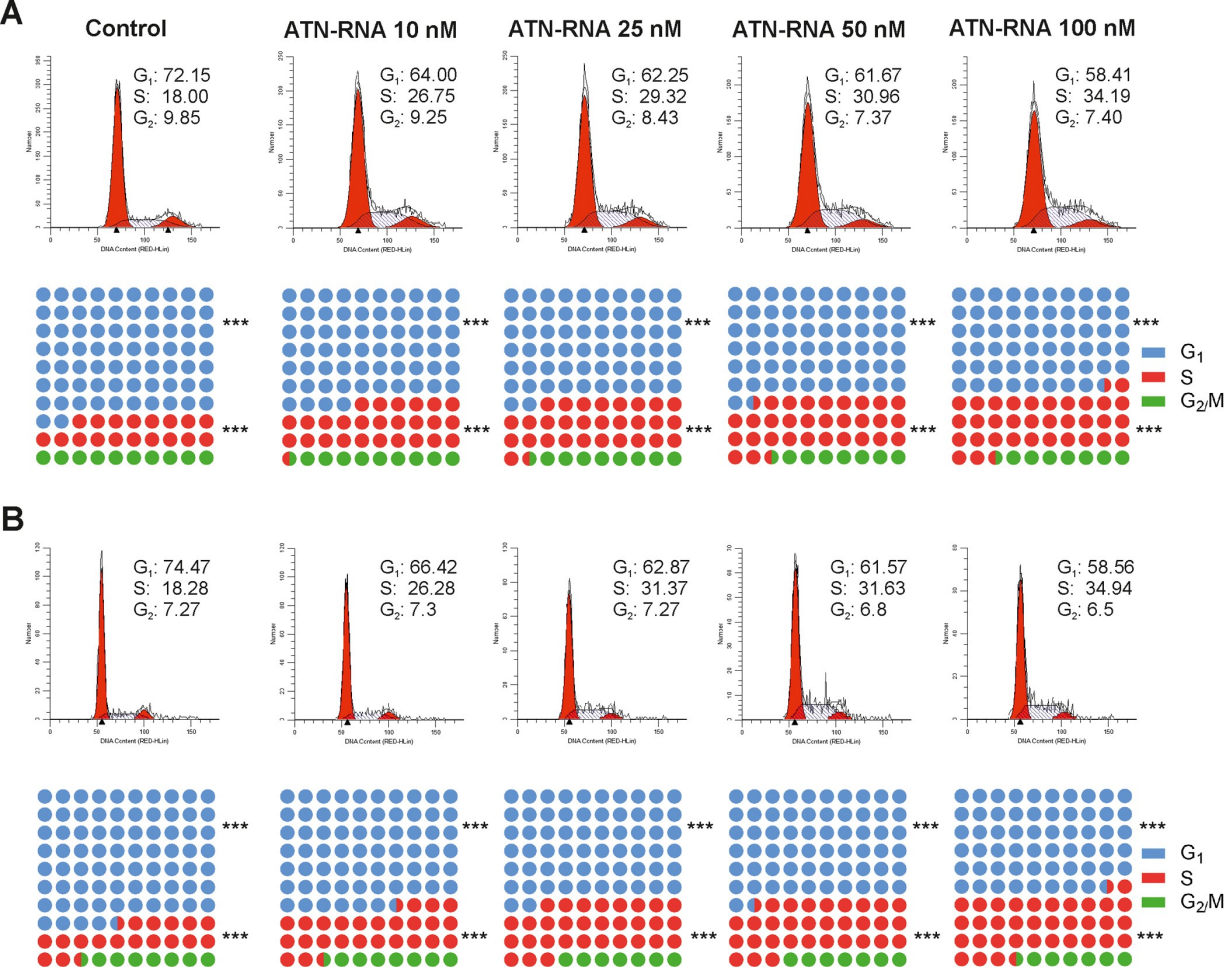
Effect of ATN-RNA on breast cancer cell cycle phase analysis. MDA-MB-231 cells were transiently transfected with increasing concentrations of ATN-RNA (from 10 to 100 nM) or scrambled siRNA (C-control) for 24 **(A)** and 48 h **(B)**. The cells were then fixed and added with Propidium Iodide (PI)/RNase A staining solution and analyzed in Muse^®^ Cell Analyzer using ModFit LT^TM^ software. A percentage of cell distribution in each cell cycle phase was summarized and shown. The cell cycle distribution profile image is shown as a representative result of three independent experiments.

To determine additionally the effect of ATN-RNA on the cell cycle progression, the MDA-MB-231 cells were treated with different concentrations of ATN-RNA for 24–48 h, and the cell cycle analysis was assessed by Muse^®^ Cell Analyzer. Cells transfected with ATN-RNA showed the cell cycle distribution with the concentration-dependent decrease in cell number in G_0_/G_1_ phase, increased in S phase and unchanged in G_2_/M phase compared to the cells transfected with unspecific control RNA (Fig 5A). ATN-RNA impacted the cell cycle by almost doubling the cells S-phase fraction from 18% to 34% for the highest ATN-RNA concentration, thus resulted with the cells arrest in S phase. The cell cycle analysis proved the non-toxic effect of ATN-RNA, since we did not observe the increase in G_1_ population. S phase arrest persists following up to 48 h of ATN-RNA treatment (Fig 5B).

**Fig 6:**
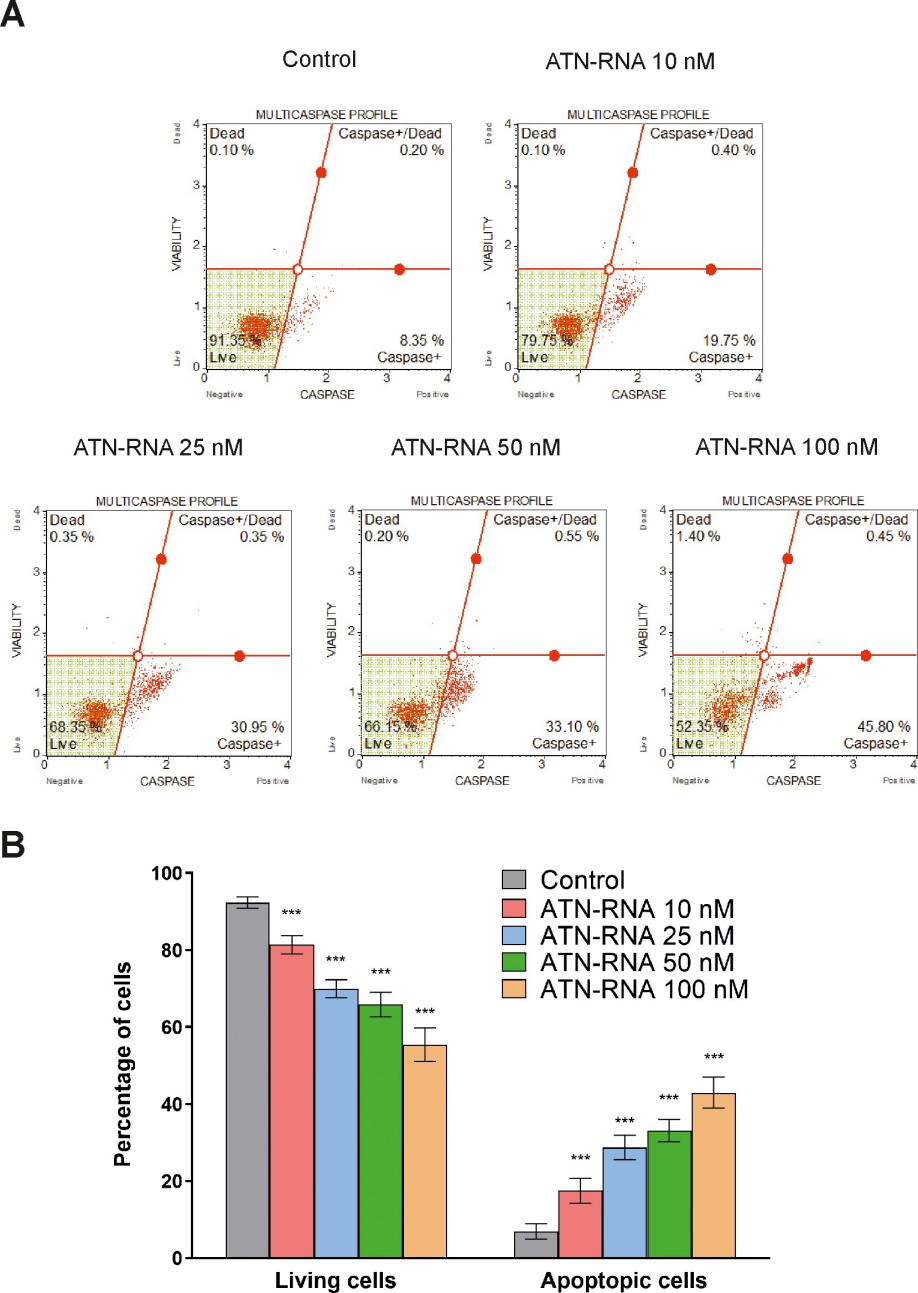
Effect of ATN-RNA on multiple caspase activation (caspase-1, 3, 4, 5, 6, 7, 8, and 9) in MDA-MB-231 cell line. Breast cancer cells were transiently transfected with increasing concentrations of ATN-RNA (from 10 to 100 nM) or scrambled siRNAs (C-control) for 24 h. The transfected cells were then incubated with Muse^®^ MultiCaspase reagent followed by analysis of the percentage of cell population in live, caspase+, caspase+/dead and dead in Muse^®^ Cell Analyzer. **(A)** The percentage of live, caspase+, caspase+/dead and dead cells profile image is shown as a representative result from one of three independent experiments. **(B)** The graphical representation of percentage of live and exhibiting caspase activity cell population transfected with ATN-RNA and scrambled siRNAs. Statistical evaluation of ATN-RNA *versus* scrambled cells treated with scrambled siRNAs was performed using one-way ANOVA followed by Tukey’s post-hoc test. Significance value: *** *P* < 0.001 compared to scrambled control (C-control). Error bars represent SD.

Thus, to examine whether ATN-RNA-induced apoptosis would be associated with the caspases activation, the expression levels and activity of caspases such as: caspase-1, -3, -4, -5, -6, -7, -8 and -9 in the ATN-RNA-treated MDA-MB-231 cells were assessed using Muse^®^ Cell Analyzer. As shown in Fig 6A, breast cancer cells treated with ATN-RNA exhibited enhanced multi-caspase activity in a concentration-dependent manner. The multi-caspase activity was 17.48 ± 3.22, 28.73 ± 3.15, 33.15 ± 2.90 and 42.95 ± 4.03% respectively, at 10; 25; 50 and 100 nM ATN-RNA concentration compared with the control (Fig 6B). Thus, we observed almost 5-fold increase of the population of the apoptotic cells with the lowest ATN-RNA concentration, whereas almost 12-fold with the highest one.

### ATN-RNA has an impact on spheroids integrity

**Fig 7:**
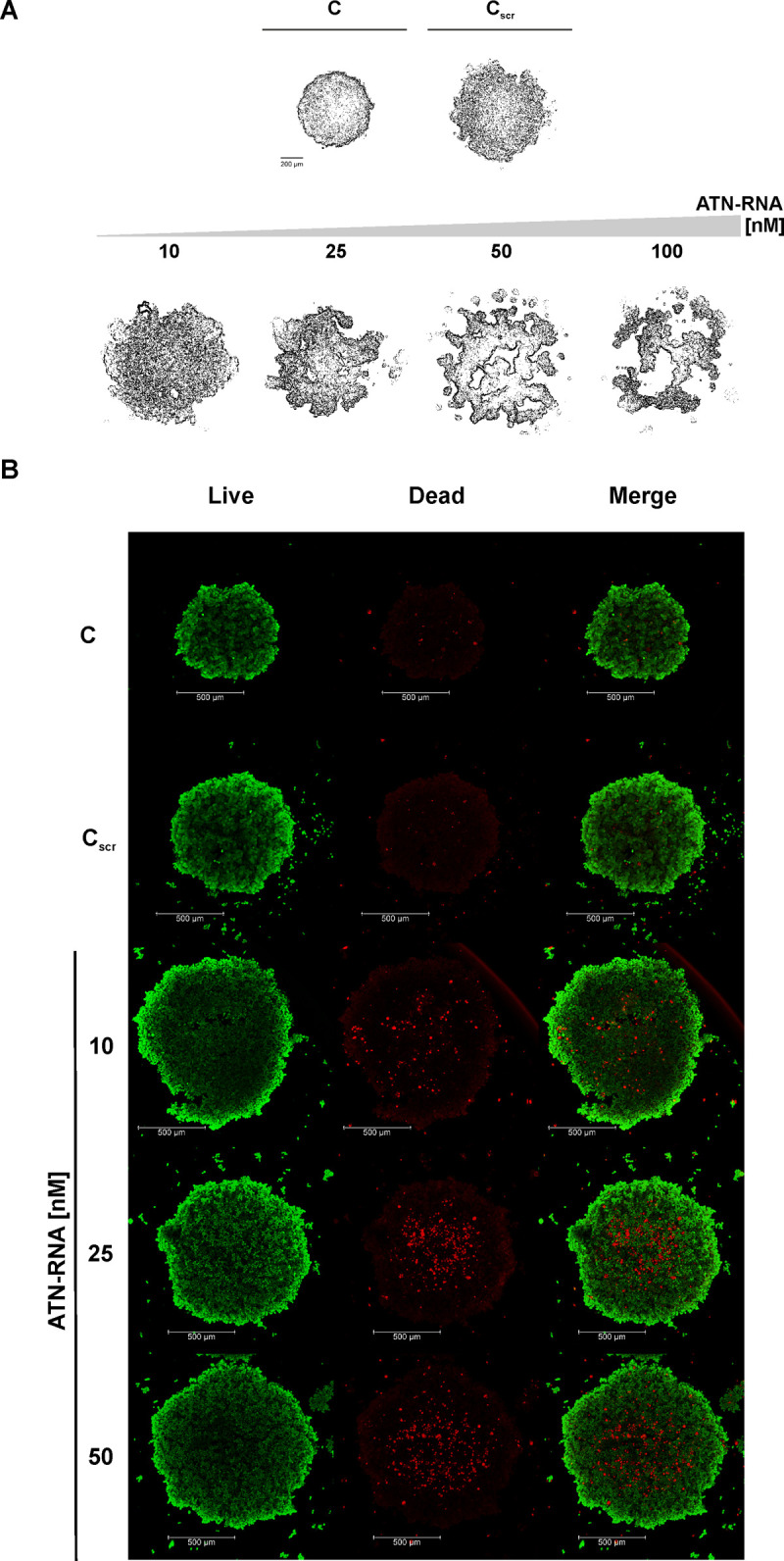
Effects of ATN-RNA on viability and spheroid structure in MDA-MB-231 cells. **(A)** Monolayer cultures were transfected with indicated amounts of ATN-RNA oligonucleotides and after 24 hrs, cellular spheroids of MDA-MB-231 cells were generated from 5000 cells in Perfecta3D^®^ 96-well Hanging Drop Plate and cultured for up to 120 hours. Scale bars, 200 μm. Scrambled siRNAs (C_scr_). **(B)** The viability of the ATN-RNA transfected cells within spheroids using LIVE/DEAD Cell Imaging Kit. Left and middle panels present live cells (green) and dead cells (red), respectively. The right panels show merge of two fluorescent images. Scrambled siRNAs (C_scr_), 10, 25, 50 concentrations of ATN-RNA used for transfection. Fluorescence images were taken using Leica TCS SP5 confocal laser scanning microscope and Plan Apo 63×1.4 NA oil-immersion objective. Scale bars, 500 μm.

To visualize the involvement of TNC in tumor formation, the 3D culture model was applied. Since 3D cell culture models mimic better the *in vivo* behavior of cells in tumour tissues and are excellent surrogates to predict tumorigenic potential *in vivo*, the ability to spheroid maintenance of breast cancer cells was assessed after ATN-RNA treatment. We have observed that down-regulation of TNC led to the disintegration of the spontaneously forming spheroids of MDA-MB-231. The clearly visible effect on the spheroid viability was observed even with the lowest ATN-RNA concentration. The increased concentrations of dsRNA had a great impact on spheroids integrity, resulting in structure disintegration at the highest concentration of 100 nM (Fig 7A). The ATN-RNA application influenced the spheroid volume and shape, displaying the total shrinking of the compact structure into the small fragments with the highest ATN-RNA concentration (Fig 7A).

To have a better insight into the MDA-MB-231 spheroids structure and the ATN-RNA impact on their viability, the confocal microscopy imaging was assessed. The analysis of fluorescent labelling with green-fluorescent calcein-AM of living and dead cells within the spheroid with the Live/Dead Viability/Cytotoxicity Kit was carried out. As revealed by the image of the untreated MDA-MB-231 cells, compact multicellular spheroids were obtained. Fluorescence images revealed the overall morphology of the MDA-MB-231 spheroids (Fig 7B). The cell density in the core of the untreated spheroid was high and no dead cells were identified. The similar pictures were obtained for the control. Furthermore, all conditions with different ATN-RNA concentrations resulted with losing the spheroid density, increased dimensions and appearing a higher population of dead cells. It is worthy of note, that the spheroids treated with increasing ATN-RNA concentrations did not display a smooth contour following 24 h of treatment and subsequently, their round shape was markedly altered by the treatments. After the treatment with 50nM concentrations, the spheroids showed the strong overrepresentation of dead cells (Fig 7B). Thus, the 100nM concentration of ATN-RNA was most likely too high for the cells viability and we were not able to keep the spheroids in shape, that would allow for the imaging.

### TNC is involved in EMT processes

**Fig 8:**
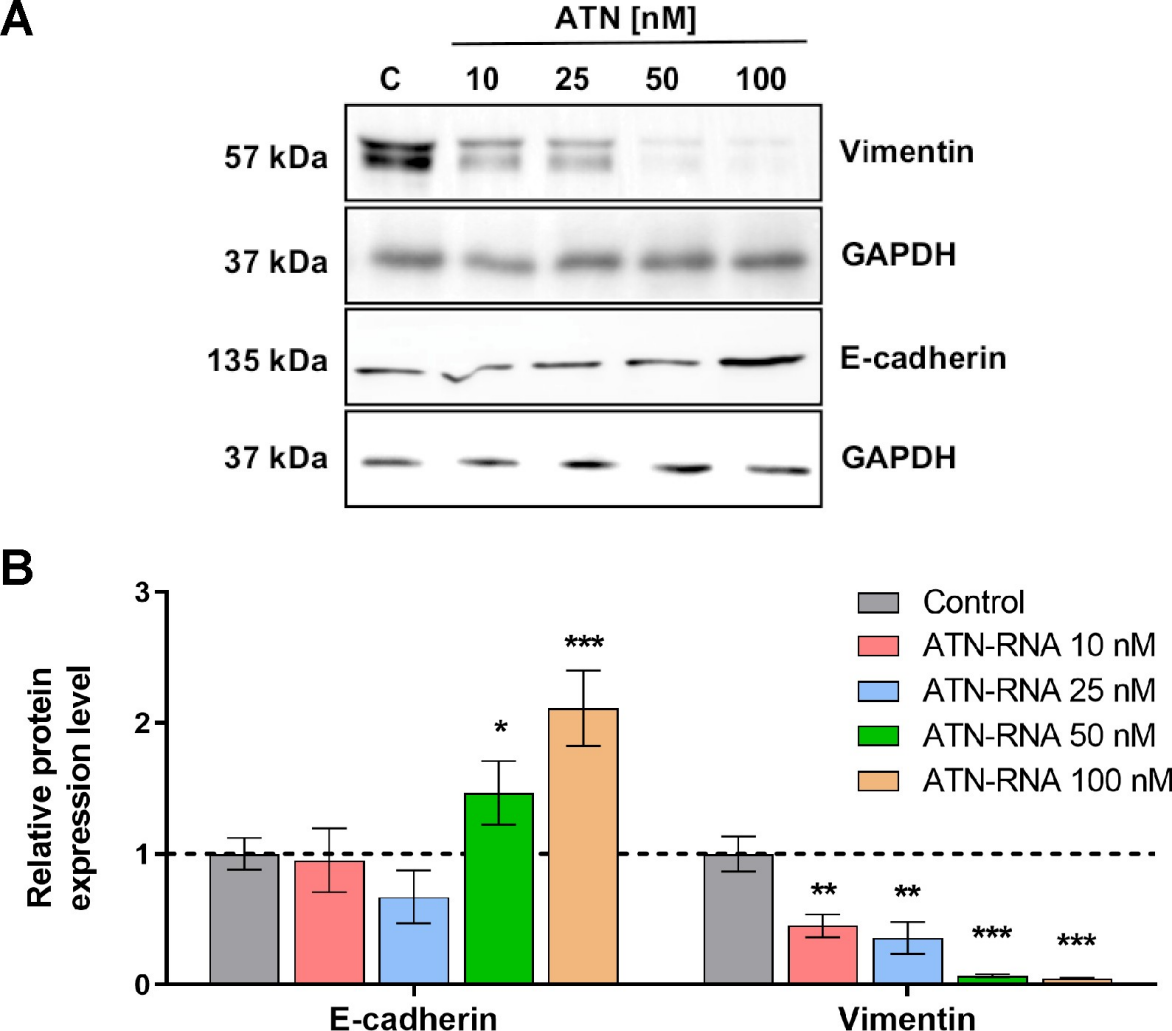
The effect of TNC down-regulation on EMT process of MDA-MB-231 cells. **(A)** The protein expression levels of E-cadherin, vimentin and GAPDH. **(B)** Western blot analysis of ATN-RNA effects on the EMT process revealed a significant increase in E-cadherin level followed by the drop of the expression of vimentin protein. The data represents the means ± SD from 3 independent experiments.

As the consequence of these finding we analyzed the expression level of proteins involved in EMT processes. We took into account two main EMT markers, E-cadherin and vimentin. Western blot analysis shows the significant increase of E-cadherin level followed by the drop of the expression of vimentin protein (Fig 8A). These observations were concentration-dependent, showing the highest efficacy for ATN-RNA at the concentration of 100nM for E-cadherin expression. Similarly, for vimentin we have observed the highest decrease of expression upon ATN-RNA transfection at 100nM concentration (Fig 8B).

## Discussion

TNC is the main ECM protein of various tumors and its over-expression is repeatedly observed in breast cancer cells both *in vitro* and *in vivo*, indicating a role for this extracellular matrix glycoprotein in neoplastic pathology. Moreover, its high expression correlates with worsened patient survival prognosis in several cancer types [37]. In breast cancers, several studies demonstrate that high expression of TNC is not only an indicator of poor prognosis, but also correlates with metastasis to distinct organs such as lymph nodes, liver and lung [38–40]. TNC plays a substantial role in EMT, that is believed to be a key mechanism in cancer progression whereby cancer cells acquire more aggressive behavior [4, 39]. In human breast cancer specimens, TNC is co-expressed with the mesenchymal marker vimentin [41]. The mechanistic role of TNC in the process of EMT remains poorly defined, however, studies suggest that TNC can induce an EMT like phenotype in MCF-7 breast cancer cells *via* the αVβ6 and αVβ1 integrins [42, 43]. Many studies on various cancer tissues have demonstrated down-regulation of epithelial markers including E-cadherin, plakoglobin and cytokeratin, as well as the up-regulation of mesenchymal markers such as N-cadherin and vimentin and expression of EMT transcription factors SNAI 1/2 and TWIST. Since these changes towards mesenchymal phenotype could correlate with invasiveness, metastatic potential and poor patient’s outcome, we have investigated the effect of TNC knockdown on the expression levels of EMT markers. Our results show that down regulation of TNC reverses the malignant phenotype of the cancer cells. As the experimental result we observed the down-regulation of mesenchymal marker—vimentin followed by the up-regulation of epithelial marker—E-cadherin. This indicates ATN-RNA as a potential therapeutic agent, which could switch the mesenchymal phenotype of breast cancer cells to the epithelial one, inhibiting the ability to metastasis and invasion. Additionally, it has been also shown that TNC as the ECM component plays also a role in cell to cell or cell-matrix attachment most probably inhibiting the cells’ migration. In approach with ATN-RNA, it seems that TNC in breast cancer cell line plays an anti-adhesive role, which would affect the cell migration and invasion ability in addition to EMT processes. Thus, TNC down-regulation seems to enhances the adhesiveness of cancer cells, showing the direct involvement of TNC in cell adhesive properties. Targeting the TNC in potential therapy might be also highly beneficial since it has been already established, that TNC maintain a stem cell niche in the brain tumors, thus could promote the tumor cell invasion. Therefore, its overexpression largely contributes to radio/chemotherapy resistance and tumor recurrence. In fact, it has been shown that targeting GBM invasion increases tumor sensitivity to temozolomide [44]. TNC also promotes stromal events such as the angiogenic switch and the formation of more but leaky blood vessels involving Wnt signaling and inhibition of Dickkopf1 (DKK1) in a neuroendocrine tumor model [45].

We observed significant ATN-RNA–mediated down-regulation of TNC was in concordance with the observed changes in proliferation and migration rates. The results show that the ATN-RNA transfected cells lose their ability to migrate, thus showing the involvement of TNC in breast cancer invasiveness. Our data indicate also that, the down-regulation of TNC expression in MDA-MB-231 cancer cell lines inhibits proliferation along with induction of cell death. We were able to determine the TNC impact on the apoptosis by measuring level of both caspases initiating intracellular events (caspase-2, -8, -9) and effector caspases-3, -6 and -7.

Orend et al. [46] demonstrated that TNC causes cdk2 inactivation and blocks cell cycle progression from G_1_ phase to S phase of anchorage-dependent fibroblasts by interfering with fibronectin–syndecan-4 interactions. For the proliferation of anchorage-dependent cells, attachment to the ECM is required. Detachment of fibroblasts by a pure tenascin-C substratum results in G_1_-phase arrest by inactivation of the cdk2 complex in a CKI-dependent manner [46]. In contrast to fibroblasts, proliferation of most tumor cells is stimulated by TNC [47]. This shows that the effect of TNC on proliferation is cell type-specific and suggests that in cancer cells, the cdk2 complex is not repressed in the presence of TNC. In leukemia, breast carcinoma and glioma were found subpopulations of stem-like cells that support tumor growth [48]. In such cells, adhesion on the TNC substratum may override the G_0_/G_1_ and G_l_/S cell cycle checkpoints, which may explain the increased proliferation rate [47]. The G_1_/S transition is enforced by cyclin E/cdk2 activation *via* syndecan-4 related signalling. It has been shown that TNC in tumor cells binds to the syndecan-4 binding site in fibronectin, thereby blocking syndecan-4 ligation, releasing the tumor cells from the suppressive effect of fibronectin on their proliferation [49]. This would appear to indicate that TNC indirectly blocks some signalling pathways by inactivating syndecan-4 and activating cdk2. In the present study, we observed the induced accumulation of cells in S-phase with a concomitant decrease in number of cells in G_1_-phase. Since this phenomenon was not observed in untreated cells, we concluded that the accumulation of cells in S-phase was largely attributed to the presence of ATN-RNA. The dose-related S-phase cell cycle accumulation described here appears to be primarily the result of TNC down-regulation. Since, inhibitory phosphorylation of cdk2 is maximal during S- and G_2_-phase it is likely that decreased level of TNC caused by ATN-RNA negatively affects cdk2 activation in S-phase and inhibition of the cell cycle in this phase. However, the exact mechanism should be the subject of future investigations. It seems likely that, down-regulation of TNC mRNA may be a unique approach to sensitizing cancer cells with S-phase chemotherapy. It has been already shown, that S-phase cell arrest can be impacted by the potential therapeutic agent incorporation into DNA during replication e.g. nucleosides analogous [50–52]. Mechanistically, cell cycle analysis revealed that ATN-RNA reduces cell proliferation primarily *via* cell cycle arrest in S-phase. The desirability of targeting S-phase as a mode of action of breast cancer therapeutics is underlined by the topoisomerase I (TOP1) inhibitor irinotecan, which is a clinically effective pharmaceutical for advanced and metastatic breast cancer [53].

Since we used RNAi technology and dsRNA for TNC downregulation that as has been shown may also induce various sequence-dependent and sequence-independent events including immune response activation, we have also looked at the expression level of the genes possibly stimulated by the dsRNA. The stimulation of cytoplasmic IFN-inducible dsRNA-activated protein kinase (PKR), retinoid acid-inducible gene-I (RIG-I), 2′-5′-oligoadenylate synthetase (OAS), interferon gamma inducible factor 16 (IFI16) and endosomal Toll-like receptors TLR3, TLR7, TLR8 leads to the activation of interferon regulatory factors and secretion of type I and III interferons. These molecules *via* autocrine/paracrine signaling activate (STAT) signaling pathway and lead to sequence-independent changes in cell division, growth or apoptosis [54–56].

However, our results did not show the ability to induce the immune response in breast cancer cell line at the level of the selected genes expression. Thus, our collected data of apoptosis and cell cycle analysis, together with the immune response analysis allowed us to hypothesize that ATN-RNA is specifically involved in apoptosis induction, without possible off-target effect and immune response activation.

Closely mimicking the tumor environment 3D cell line spheroids model of breast cancer confirmed TNC direct involvement in tumor spheroids viability, thus the down-regulation of this protein results in disintegration of spheroid morphology. One can however observe, that the application of 100 nM concentration of ATN-RNA in some cases impacts the unexpected changes and the discrepancy regarding to lower concentrations. 100nM ATN-RNA concentrations seems to be too high for the viability of cells resulting with the more likely unspecific effects. This would suggest, that the dose of ATN-RNA applied to cancer cells must be then carefully established to avoid any toxic or unspecific effects. In our approach we established then the TNC loss-off-function MDA-MB-231 cell line, what resulted in a response to abrogate potency to tumor growth. We have found that the viability of microtumor is abolished in a dose-dependent manner, especially at the tumor borders, but also with the predominant presence of dead cells in the spheroid core (Fig 8). Since a large number of dead cells was observed in all spheroids treated with ATN-RNA, we could suspect the possible diffusion of the ATN-RNA agent and high levels of cellular stress inside the 3D structure.

Our results share a number of similarities with Xia et al.’s [57] findings with glioma “go-or-grow” phenomenon which represents characteristic, fast growing tumor cores and diffuse tumor borders with a low proliferation rate. The cellular mechanisms of this phenotypic switch in intracranial tumor xenografts are associated with decreased tumor invasion and increased tumor proliferation due to decreased TNC level in tumor microenvironment [57]. Taken together our observations, we conclude that, even when we observe the cells diffusion from the periphery of the spheroids, it more likely cannot be connected to the possible invasive cells potential.

There is a large body of literature that highlights the role of TNC in tumor expansion and metastasis [46, 47, 49, 58, 59]. Although, participation in pathological processes of other tenascin family proteins, especially tenascin-X (TNX, encoded by the *TNXB* gene) is still controversial. Liot et al. [58] conducted a large meta-analysis using the Gene Expression Omnibus and The Cancer Genome Atlas databases and immunohistochemistry staining of Tissue MicroArrays to analyze TNX expression in 13 types of cancer in the context of tumor progression. Their results show that TNX mRNA and protein levels are down-regulated in most cancers, except for glioma. Discrepancies between gene and protein expressions were observed for stomach adenocarcinoma, ovary cancer and malignant mesothelioma. Survival differences between the established *TNXB*^low^ and *TNXB*^high^ subgroups were evaluated with Kaplan–Meier survival curves. As expected, the expression level of *TNXB* mRNA were significantly associated with the clinical outcomes of lung adenocarcinoma (*P* = 0.0014) and breast carcinoma (*P* = 0.0234) patients. These results indicate that high expression of *TNXB* correlates with good prognosis and survival rate in the breast and lung carcinomas which have the highest incidence and mortality in the world among all of cancers.

Our experimental data are in concordance with the oncogenomic portals deep analysis showing the association of the TNC over-expression and the poor survival of the breast cancer patients. The analogous analysis of the TNX expression level shows the opposite effect on the survival. The over-expression of *TNXB* mRNA correlates with the long survival of the breast cancers patients, whereas its down-regulation is linked to shorter time of survival. The finding is consistent with previous research by Matsumoto et al. [60], which found that the absence of TNX may promote tumor cell behaviour. In particular, B16-BL6 melanoma cell growth and metastasis was increased in tenascin-X double knockout mice (TNX-/-) in comparison to littermates. Cells in natural state express TNX reciprocal to TNC, so therapy based on silencing expression should target only one of those proteins to not disturb functionality of the second one. The alignment of these two ECM proteins—TNC and TNX shows that they share only a limited number of nucleotides, avoiding the ATN-RNA sequence overlap with the *TNXB*. Therefore the ATN-RNA therapeutic tool only matches to the *TNC* sequence (Fig 2A). The down-regulation of TNC with ATN-RNA prolongs then patients’ survival protecting the suppressive activity of TNX. Regarding the presence of the TNC isoforms, almost universal and high level of expression of all these isoforms in breast carcinomas coupled with their tumor-restricted distribution make them still a plausible therapeutic target for ATN-RNA, since the sequence of ATN-RNA is directed to the EGF–like domains present always in all splicing variants.

Looking at the data, one can however see the discrepancy between gene and protein expression of the isoforms at the breast and breast cancer tissue level in our analysis. The repositories in the data bases allowed us to clearly show the expression of the TNC and TNX at the mRNA level which was detected for 1085 patients. The Human Protein Atlas at the same time shows the data for only 11 breast cancer patients at the protein level. Thus, we assume the discrepancy might arise from the highly different number of the samples with the relevant data. The data on the mRNA and protein level, although differ in the number of the samples, still keep, in our mind, the tendency which shows the overexpression of TNC in breast cancer samples.

Additionally, the performed analysis including the TNC impact on the patient survival, with the data for 196 breast cancer patients, provides, in our mind, strong premise for considering the TNC as the good candidate as the therapeutic target and the malignancy predictor for breast cancer patients.

Our results as well as the oncogenomic data, both correlate TNC expression with the poor patients survival and enhance the utility of TNC as a kind of universal therapeutic target for other tumors types, where TNC overexpression is associated with poor prognosis.

However, we also emphasize the fact that no ECM protein exists in isolation and suggest that, even targeting such potent molecule as TNC alone, may not be sufficient to completely damage the neoplasm and benefit patient outcome. Therefore, targeting TNC may be potentially supportive in designing more effective anticancer treatments when used in conjunction with radiotherapy, and chemotherapy.

## Materials and methods

### Cell culture conditions

Human MDA-MB-231 breast cancer cells triple negative–ER-; PR-; HER2- were purchased from ATCC (American Type Cell Collection, Manassas, VA, USA). Cells were maintained in DMEM medium supplemented with 10% foetal bovine serum (FBS), 100 U/ml penicillin and 0.1 mg/ml streptomycin (all from Sigma-Aldrich, MO, USA). Cell line was cultured in humidified incubator with 5% CO_2_ at 37°C.

### dsRNA preparation

ATN-RNA was prepared as previously described [25, 61]. Briefly, ATN-RNA was generated by *in vitro* transcription of ATN-DNA flanked by T7 and T3 RNA polymerase promoter regions. The two strands were synthesized individually, purified, and then added together for annealing and renaturation (50 mM Tris-HCl pH 7.5, 50 mM NaCl, 95°C for 3 min, 75°C for 30 min and slow cooling down for 4 h to 25°C) [62]. Hybridization was analysed on 6% polyacrylamide gel electrophoresis with 7M urea.

### *In vitro* transfection

Transfection of dsRNA was performed with a commercial reagent, Lipofectamine^®^ 2000 (Thermo Fisher Scientific, MA, USA) in 24-well plates according to manufacturer’s instructions. Briefly, the day before transfection, confluent layers of cells were trypsinized, counted and resuspended. Cell suspension was plated into each well of the 24-well plates, so that they could become about 70% confluent next day at the time of transfection. The cells were then incubated with dsRNA (10; 25; 50 and 100 nM–final concentration) for 48 h.

### 3D spheroid culture

MDA-MB-231 cells were initially maintained as a monolayer culture and transfected with 10; 25; 50; 100 nM (final concentration) of ATN-RNA and Lipofectamine^®^ 2000 (Invitrogen, CA, USA). After 24 h cells were seeded in Perfecta3D^®^ 96-well Hanging Drop Plate (3D Biomatrix, Ann Arbor, MI, USA). Spheroids in hanging drops were formed by pipetting 5×10^3^ cells in 50 μl of complete growth medium. The cell culture medium was supplemented on the third day of culture by adding 7 μl fresh growth media to each drop. Spheroids were grown for 5 days.

### RNA isolation

Total RNA from breast cell lines was isolated with TRIzol^®^ Reagent (Thermo Fisher Scientific, MA, USA), then purified using the Ambion^®^ DNA-*free*^TM^ Kit (Applied Biosystems, CA, USA) according to the manufacturer’s instructions. The reverse transcription was carried out using 1 μg RNA, random primer and RevertAid^TM^ H Minus M-MuLV reverse transcriptase (Thermo Fisher Scientific, MA, USA).

### Quantitative RT-PCR

Real-time qPCR was performed to assess transcripts of TNC (Tenascin-C; TNC-R:GGGATTAATGTCGGAAATGGT, TNC-L: CCGGACCAAAACCATCAGT), OAS1 (2'5'-oligoadenylate synthetase 1; OAS1-R: CAGGAGCTCCAGGGCATAC, OAS1-L: CATCCGCCTAGTCAAGCACT), OAS3 (2'-5'-oligoadenylate synthetase 3; OAS3-R: ACGAGGTCGGCATCTGAG, OAS3-L: TCCCATCAAAGTGATCAAGGT), RIG1 (retinoic acid-inducible gene I; RIG1-R: TTGGTATCTCCTAATCGCAAAAG, RIG1-L: GGCAAGTCCCGCTGTAAAC), IFI16 (interferon gamma inducible factor 16; IFI16-R: TTTGGATGCTCTGGTCATCTT, IFI16-L: GGCATTTTGAAGAATTGGAAAG), TLR3 (Toll-like receptor 3; TLR3-R: ATCTTCCAATTGCGTGAAAAC, TLR3-L: TGGATATCTTTGCCAATTCATCT) expression relative to HPRT (hypoxanthine phosphoribosyltransferase; HPRT-R: CGAGCAAGACGTTCAGTCCT, HPRT-L: TGACCTTGATTTATTTTGCATACC), ACTB (β-actin; ACTB-R: CCAGAGGCGTACAGGGATAG; ACTB-L: CCAACCGCGAGAAGATGA) using the thermocycler LightCycler^®^ 480 (Roche Applied Science, Germany). The reaction mixture was prepared with the LightCycler^®^ 480 Probes Master Kit (Roche Applied Science, Germany) and included 1x Master Mix, 0.1 μM of probes, 1 μM of each primer, 1 μl of template cDNA and water to a final volume of 10 μl. The PCR conditions for all genes were as follows: initial incubation step at 95°C for 5 min followed by 45 cycles of amplification for 15 s at 95°C, 30 s at 55°C and 30 s at 72°C. the final cooling step was 40°C for 30 s. All standard curves were generated by amplifying a series of 2-fold dilution of cDNA.

### Protein isolation

MDA-MB-231 3D spheroid cells treated with ATN-RNA were sonicated in 50 mM Tris-HCl buffer pH 7.5 with 1x protease inhibitors cocktail (Sigma-Aldrich, MO, USA) for 3×15 s and centrifugated 5 min at 10000 rpm. Supernatant containing soluble proteins was spectrophotometrically measured at a wavelength of 280 nm to assess the protein concentration and then used for Western blot analysis.

### Western blot analysis

100, 50 and 10 μg of protein extracts were separated by 15% SDS-PAGE for TNC, EMT proteins and GAPDH, respectively. PageRuler™ Plus Prestained Protein Ladder (Thermo Fisher Scientific, MA, USA) was used as the size marker. The electrophoresis was run at a current of 30 mA and a voltage of 250 V. Transfer was carried out for 30 min at a current of 130 mA and a voltage of 100 V in wet transfer blotter (Bio-Rad, CA, USA) onto PVDF membrane of 0.45 μm pore size (GE Healthcare, IL, USA) in transfer buffer (25 mM Tris base, 190 mM glycine, 20% methanol). Blots were rinsed once in PBST20 (PBS, 0.05% Tween 20). The membranes were placed in the SNAP i.d.^®^ 2.0 apparatus (EMD Milipore, MA, USA), where it was blocked for 10 min with a 0.5% solution of skimmed milk in PBST20 (Sigma-Aldrich, MO, USA). Hybridization was done with polyclonal Tenascin-C antibody (Santa Cruz Biotechnology, TX, USA), E-cadherin and vimentin from the Epithelial-Mesenchymal Transition (EMT) Antibody Sampler Kit (Cell Signaling Technology, MA, USA), while for detection of glyceraldehyde 3-phosphate dehydrogenase (GAPDH) monoclonal antibody was used (Santa Cruz Biotechnology, TX, USA). Antibodies were diluted 1:500 in 3% BSA. Membranes were incubated with the primary antibody for 10 min or overnight, depending on the level of detection of the protein then washed 3 times with PBST20. Secondary anti-rabbit IgG and anti-mouse IgG antibodies conjugated with horseradish peroxidase (HRP) were used (Sigma-Aldrich, MO, USA). Detection of proteins was carried out with WesternBright Sirius Chemiluminescent Detection Kit (Advansta, CA, USA). Intensity of individual bands was analyzed qualitatively by Multi Gauge ver. 2.0 (Fujifilm, Tokio, Japan). Final expression levels of Tenascin-C, E-cadherin and vimentin were compared to GAPDH expression level.

### Real-time proliferation assay

Experiments were carried out using the *xCELLigence* Real-Time Cell Analyzer (RTCA) DP instrument (Roche Applied Science, Germany). The system measures time-resolved electrical impedance changes resulting from cell proliferation to electrodes located on the plate surface (E-plate, ACEA Biosciences, San Diego, CA, USA). Initially, 100 μl of cell-free complete growth medium (10% FBS) was added to the wells. After leaving the 16-well E-plates at room temperature for 10 min in tissue culture hood, the background impedance for each well was measured. Transfected with 10; 25; 50; 100 and 150 nM (final concentration) of ATN-RNA, MDA-MB-231 cells were seeded in E-plates at a density of 10×10^4^ per well in complete growth medium (100 μl). After leaving the plates at room temperature for 30 min to allow cell attachment, they were placed in RTCA DP instrument and the impedance value of each well was monitored by the *xCELLigence* system and defined as a *Cell Index* value (CI), a dimensionless parameter, which was directly proportional to the total area of microelectrodes populated by the cells. Impedance was measured for 72 h. CI was monitored every 15 min during the experiment. Obtained CI values were entered to the GraphPad Prism v5.01 (GraphPad Software, La Jolla, CA, USA) software and used to calculate half maximal inhibitory concentrations (IC_50_).

### Transwell migration assay

Transwell cell migration experiments were performed using *xCELLigence* RTCA DP Analyzer in Cell Invasion and Migration (CIM) plates (ACEA Biosciences, San Diego, CA, USA) with each well consisting of an upper and a lower chamber separated by a microporous polyethylene terephthalate (PET) membrane containing randomly distributed 8 μm-pores. Prior to migration assay, MDA-MB-231 cells were transfected with ATN-RNA. Initially, 160 μl of complete growth medium (supplemented with 10% FBS) was added to the lower chamber and 25 μl of serum-free growth medium to the upper chambers of CIM-plate. To initiate a transwell migration experiment, cells were detached, resuspended in serum-free growth medium and seeded in the upper chamber at a density of 5×10^4^ cells in 175 μl per well, treated with ATN-RNA (in 10; 25; 50 and 100 nM concentration). After cell addition, CIM-plates were incubated during 30 min at room temperature in tissue culture hood to allow the cells to embed onto the PET membrane. Impedance of the microelectrode array attached to the bottom side of the microporous membrane was monitored and converted by RTCA DP software to CI. Readouts were performed every 15 min for up to 72 h. Using GraphPad Prism v5.01 obtained CI values from each experimental condition were plotted against time, fitted to four-parameter logistic non-linear regression model and the half-time of migration (half maximal effective time, ET_50_) was calculated [63].

### Adhesion assay

Cellular reattachment from suspension was monitored by the *xCELLigence* system based on RTCA DP instrument. Experiment was carried out in 16-well E-Plates VIEW (ACEA Biosciences, San Diego, CA, USA) with electrode-free viewing strip which allows for microscopy observations. MDA-MB-231 cell transfections with ATN-RNA at final concentrations of 10; 25; 50; 100 nM were done 24 h prior to the reattachment assay. 1 h before test, E-Plates VIEW were coated with 0.5% BSA dissolved in PBS, left at 37°C and 5% CO_2_. Then wells were washed with PBS and filled with 50 μl of serum-free growth medium supplemented with 0.25% BSA. Plate was next plugged in the RTCA DP instrument for first measurement of the background impedance. Afterwards the same plate was seeded at the density of 5×10^4^ cells in 150 μl medium per well. System was subsequently re-inserted into the RTCA and left for 10 min to stop movements of media in well. The impedance measurements lasted for 4 h with readout at 3 minute intervals. The resulting IC data depends on the number of adherent cells.

### Cell cycle

MDA-MB-231 cells (6×10^4^ per 6-well plate) were seeded and exposed to ATN-RNA (0; 10; 25; 50 and 100 nM) for 24 h and 48 h. The harvested cells were washed in PBS and 200 μl of Muse^®^ cell cycle reagent (EMD Millipore, MA, USA) was added. The cells were incubated for 30 min at RT in the dark. Cell cycle distribution was analyzed by Muse^®^ Cell Analyzer (Merck Millipore, MA, USA).

### Multi-caspase assay

The activity of caspase-1, -3, -4, -5, -6, -7, -8 and -9 was measured using a Muse^®^ Multi-caspase kit (MCH100109, Merck Millipore, MA, USA) according to the manufacturer's instructions. MDA-MB-231 cells were incubated with various concentrations of ATN-RNA (0; 10; 25; 50 and 100 nM) for 24 h. Cells were harvested and incubated with 5 μl of Muse^®^ Multi-Caspase reagent working solution at 37°C for 30 min. Then, 150 μl of Muse^®^ Caspase 7-AAD working solution was added to each sample. Multi-caspase assay was performed with Muse^®^ Cell Analyzer (Merck Millipore, MA, USA).

### Microscopy and image analysis

The morphology of the spheroids was visualized by a Leica DMI4000 B inverted microscope (Leica Microsystems, Wetzlar, Germany) with 5× magnification objective. For assessment of ATN-RNA effects in 3D cultures on spheroid morphology we applied author’s spheroid-edge detection method. In brief, for each ATN-RNA concentration at experimental endpoint (72 h) high magnification microscopy images were taken with a Leica Application Suite (Leica Microsystems, Wetzlar, Germany) with a 1 ms exposure time. Central micro tumours were cropped into the final images for spheroid-contour processing. The size of each cropped image in all experiments was 567 x 567 pixels (24 Bit Color RGB), with resolution of 96 dpi. In next step, the contour of spheroids was identified, followed by *Contour Tool* in Corel^®^ PHOTO-PAINT X5 (Corel Corporation, Ottawa, ON, Canada). The size of the spheroids was analysed using the analysis tools in Photoshop CS5 software (Adobe Systems, San Jose, CA, USA).

### Viability analysis of spheroids

The viability of MDA-MB-231 spheroids treated with 10; 25 and 50 nM of ATN-RNA was analyzed using LIVE/DEAD Cell Imaging Kit (488/570) (Thermo Fischer Scientific, MA, USA). MDA-MB-231 cells were transfected with ATN-RNA and seeded onto 96-well U-bottom plates at a density of 5×10^3^ cells/well in the 100 μl of DMEM growth medium supplemented with 10% of FBS and 1% of antibiotic solution and cultured at 37°C and 5% CO_2_ saturation. After 3 days, spheroids were replaced into prewarmed (37°C) fresh medium containing LIVE/DEAD reagent (calcein-AM/ethidium homodimer 1) prepared according to the manufacturer’s protocol and incubated for 30 min under growth conditions. Next spheroids were moved to the glass-bottom dishes and placed in FluoroBrite DMEM (ThermoFisher Scientific). Life-cell imaging was done using Leica TCS SP5 confocal laser scanning microscope equipped with White Light Laser (470–670 nm) and environmental cell culture chamber provides controlled conditions of temperature, CO_2_ saturation and humidity. Sequentially scanned images were collected at Ex: 488 nm/Em: 515 nm for live cells and Ex: 570 nm/Em: 602 nm for dead cell, respectively, using a Plan Apo 63×1.4 NA oil-immersion objective. Image processing and analysis was done in Leica LAS AF software.

### Databases

The expression data of *TNC*, *TNN*, *TNR* and *TNXB* genes were retrieved from The Cancer Genome Atlas (TCGA) and the Human Protein Atlas. The GEPIA (Gene Expression Profiling Interactive Analysis) online web server (http://gepia.cancer-pku.cn/) [64] collects data from the database of TCGA. The tenascin genes expression data from TCGA were retrieved using the GEPIA program. mRNA levels (log_2_ TPM+1) of tenascin genes were replotted. The survival analyses of the cancer patients with high and low levels of *TNC*, and *TNXB* expression were performed with the use of datasets and tools available in the PPISURV portal (http://www.bioprofiling.de/GEO/PPISURV/ppisurvD.html) [65]. The data for analysis of the relationship between copy number category and expression level of TNC were obtained from cBioPortal for Cancer Genomics (Memorial Sloan-Kettering Cancer Center, New York, NY, USA; http://www.cbioportal.org/) [66, 67]. Sequence alignment of tenascin-C versus tenascin-X with their relation to ATN-RNA was performed with BLASTN 2.6.1+ [68]. Accession numbers for analysed genes used in this alignment: Homo sapiens tenascin-C (TNC), mRNA—NM_002160.3; Homo sapiens tenascin-X (TNXB-S), transcript variant XB-S, mRNA—NM_032470.3; Homo sapiens tenascin-X (TNXB-L), transcript variant XB-L, mRNA—NM_019105.6. Immunohistochemical staining of tumor and normal tissues were retrieved from the Human Protein Atlas. Samples with different scales of protein staining in tumor or normal tissues were counted.

### Statistical analysis

Statistical analyses were performed using GraphPad Prism v5.01. Data was collected as triplicate from at least 3 independent experiments. The results were shown as mean ± standard deviation (SD). Differences between the means of treatments were evaluated using one-way analysis of variance (ANOVA) followed by Tukey’s test. The half-maximal inhibitory concentrations (IC_50_) were calculated by fitting experimental values to a sigmoidal bell-shaped equation. Statistical significance was designated as * *P* < 0.05, ** *P* < 0.01 and *** *P* < 0.001.

## Supporting information

S1 File (DOCX)

S1 Raw images (PDF)
