# Supplementary material for: Down-regulation of tenascin-C inhibits breast cancer cells development by cell growth, migration, and adhesion impairment
Source: PLoS One. 2020 Aug 20;15(8):e0237889. doi: 10.1371/journal.pone.0237889 (PMC7440653; doi:10.1371/journal.pone.0237889)
Supplement: S1 File — (DOCX) [file pone.0237889.s001.docx]

**Down-regulation of tenascin-C inhibits breast cancer cells development by cell growth, migration, and adhesion impairment**

Dariusz Wawrzyniak^1^, Małgorzata Grabowska^1^, Paweł Głodowicz^1^, Konrad Kuczyński^1,2^, Bogna Kuczyńska^1^, Agnieszka Fedoruk-Wyszomirska^3^ and Katarzyna Rolle^1*^

^1^ Department of Molecular Neurooncology, Institute of Bioorganic Chemistry of the Polish Academy of Sciences, Poznan, Poland

^2^ NanoBioMedical Centre, Adam Mickiewicz University, Poznan, Poland

^3^ Laboratory of Subcellular Structures Analysis, Institute of Bioorganic Chemistry of the Polish Academy of Sciences, Poznan, Poland

^*^ Corresponding author:

E-mail: kbug@ibch.poznan.pl


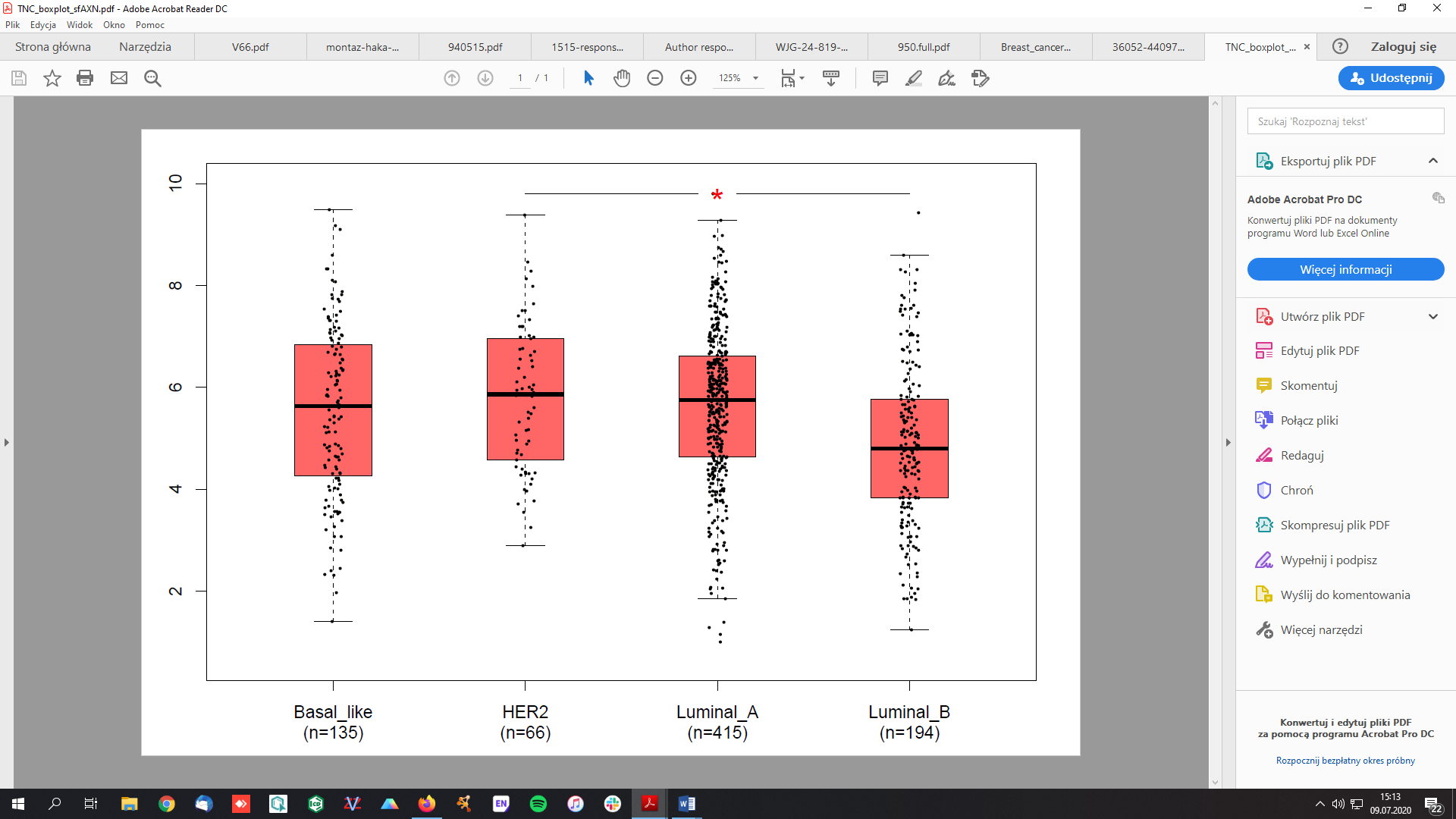


Figure A. Messenger RNA level of TNC (tenascin-C) gene in 4 subtypes of invasive carcinoma of the breast (N = 810 samples). RNA sequencing data were retrieved from the database of BRCA-TCGA and analysed using the GEPIA (Gene Expression Profiling Interactive Analysis) online web server (<http://gepia.cancer-pku.cn/>).
