# Supplementary material for: Down-regulation of tenascin-C inhibits breast cancer cells development by cell growth, migration, and adhesion impairment
Source: PLoS One. 2020 Aug 20;15(8):e0237889. doi: 10.1371/journal.pone.0237889 (PMC7440653; doi:10.1371/journal.pone.0237889)
Supplement: S1 Raw images — (PDF) [file pone.0237889.s002.pdf]

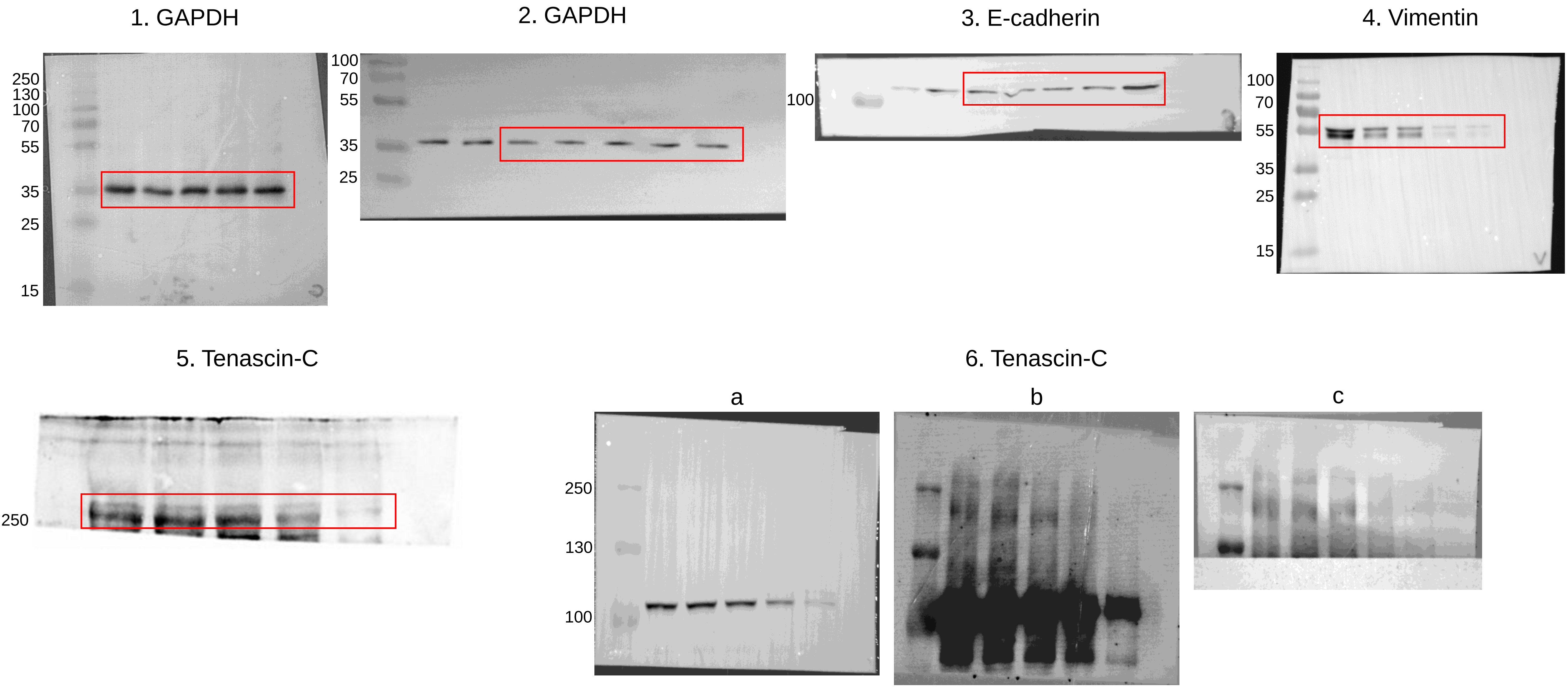

PVDF membranes used in western blot experiments. Membranes 2 and 5 are used on Figure 3. Membranes 1, 2, 3, and 4 are used on Figure 8. Samples described in the paper are marked with red frames. Due to the technical reasons Membrane 6 is presented after three different exposure times. 1 minute exposure (a) reveals about 100 kDa bands, which according to the Sofat N et al., 2011, *Tenascin-C fragments are endogenous inducers of cartilage matrix degradation*, Rheumatology International 32(9):2809-17 might represent short TNC fragment (not contained alternatively spliced domains A, B, C and D). In order to detect full-length TNC 180-250 kDa membrane was exposed for 11 minutes (b), thus showing the extremely strong signal from the lower band and visible fragment from the full-length TNC. When the lower fragment of the membrane is covered the signal from the full-lenght TNC is clearly visible (c). Therefore we decided to trim the membranes as is shown on Figure 5 to avoid the antibody binding to the shorter version of the TNC in order to obtain clear picture of the experiment.
